# Efficacy and safety of BVAC-C in HPV type 16- or 18–positive cervical carcinoma who failed 1st platinum-based chemotherapy: a phase I/IIa study

**DOI:** 10.3389/fimmu.2024.1371353

**Published:** 2024-03-28

**Authors:** Chel Hun Choi, Jeong-Won Lee, Duk-Soo Bae, Eun-Suk Kang, Duck Cho, Yong-Man Kim, Kidong Kim, Jae-Weon Kim, Hee Seung Kim, Young-Tae Kim, Jung-Yun Lee, Myong Cheol Lim, Taegwon Oh, Boyeong Song, Insu Jeon, Myunghwan Park, Wu Hyun Kim, Chang-Yuil Kang, Byoung-Gie Kim

**Affiliations:** ^1^ Department of Obstetrics and Gynecology, Samsung Medical Center, Sungkyunkwan University School of Medicine, Seoul, Republic of Korea; ^2^ Department of Laboratory Medicine and Genetics, Samsung Medical Center, Sungkyunkwan University School of Medicine, Seoul, Republic of Korea; ^3^ Department of Obstetrics and Gynecology, Asan Medical Center, Seoul, Republic of Korea; ^4^ Department of Obstetrics and Gynecology, Seoul National University Bundang Hospital, Seongnam-si, Gyeonggi-do, Republic of Korea; ^5^ Department of Obstetrics and Gynecology, Seoul National University College of Medicine, Seoul, Republic of Korea; ^6^ Obstetrics and Gynecology, Yonsei University College of Medicine, Seoul, Republic of Korea; ^7^ Graduate School of Cancer Science and Policy, National Cancer Center, Goyang, Republic of Korea; ^8^ Cellid. Inc, Seoul, Republic of Korea

**Keywords:** HPV 16, HPV 18, cervical cancer, BVAC-C, therapeutic vaccine

## Abstract

**Background:**

BVAC-C, a B cell– and monocyte-based immunotherapeutic vaccine transfected with recombinant HPV E6/E7, was well tolerated in HPV–positive recurrent cervical carcinoma patients in a phase I study. This phase IIa study investigates the antitumor activity of BVAC-C in patients with HPV 16– or 18–positive cervical cancer who had experienced recurrence after a platinum-based combination chemotherapy.

**Patients and methods:**

Patients were allocated to 3 arms; Arm 1, BVAC-C injection at 0, 4, 8 weeks; Arm 2, BVAC-C injection at 0, 4, 8, 12 weeks; Arm 3, BVAC-C injection at 0, 4, 8, 12 weeks with topotecan at 2, 6, 10, 14 weeks. Primary endpoints were safety and objective response rate (ORR) as assessed by an independent radiologist according to Response Evaluation Criteria in Solid Tumors version 1.1. Secondary endpoints included the disease control rate (DCR), duration of response (DOR), progression-free survival (PFS), and overall survival (OS).

**Results:**

Of the 30 patients available for analysis, the ORR was 19.2% (Arm 1: 20.0% (3/15), Arm 2: 33.3% (2/6), Arm3: 0%) and the DCR was 53.8% (Arm 1: 57.1%, Arm 2: 28.6%, Arm3: 14.3%). The median DOR was 7.5 months (95% CI 7.1–not reported), the median PFS was 5.8 months (95% CI 4.2–10.3), and the median OS was 17.7 months (95% CI 12.0–not reported). All evaluated patients showed not only inflammatory cytokine responses (IFN-γ or TNF-α) but also potent E6/E7-specific T cell responses upon vaccinations. Immune responses of patients after vaccination were correlated with their clinical responses.

**Conclusion:**

BVAC-C represents a promising treatment option and a manageable safety profile in the second-line setting for this patient population. Further studies are needed to identify potential biomarkers of response.

**Clinical trial registration:**

ClinicalTrials.gov, identifier NCT02866006.

## 1 Introduction

Screening programs and prophylactic vaccines have significantly reduced the incidence of cervical cancer (1). However, few treatment options are available for patients with metastatic or recurrent disease (2, 3). While adding bevacizumab to first-line combination chemotherapy can provide an overall survival advantage, treatment options after progression are limited to single-agent therapies and palliative care (4, 5). Novel therapeutic modalities are urgently needed to improve the prognosis of patients with recurrent cervical cancer.

Cancer immunotherapy has received increasing attention from clinical oncologists, and several immune checkpoint inhibitors have shown clinical activity in patients with programmed cell death ligand 1 (PD-L1)-positive cervical cancer (6, 7). Pembrolizumab combined with chemotherapy, with or without bevacizumab, improved overall survival in the metastatic first-line setting for recurrent patients compared with chemotherapy alone (8), leading to approval by the US FDA for first-line treatment. In the second- or later-line settings, pembrolizumab, nivolumab, cemiplimab, and balstilimab have shown clinical activity, further confirming the benefit of checkpoint inhibitors in the second-line setting and beyond (9–12). However, the tumor responses to these immune checkpoint inhibitors were modest. Objective response rates were less than 30%, indicating a need for more effective treatment strategies. Despite this, it’s worth noting that novel PD-1 inhibitor combination therapies have been reported. For instance, in the trial conducted by Lan et al, a PD-1 inhibitor camrelizumab plus apatinib achieved promising efficacy, with a response rate of 55.6% and a median progression-free survival (PFS) of 8.8 months (13).

Human papillomavirus (HPV) infections are the main cause of cervical cancer, and HPV E6 and E7 oncoproteins are targets for immunotherapy (14, 15). A therapeutic vaccine targeting these oncoproteins may be able to treat HPV-positive cervical carcinoma patients (16, 17). Various approaches, including chimeric virus-like particles, dendritic cell vaccines, fusion proteins, peptides, an attenuated virus, and HPV-specific T-cell therapy, have been tested (15, 18, 19).

BVAC-C is a therapeutic vaccine that utilizes CeliVax technology to activate both innate and adaptive immune effectors (20, 21). It consists of autologous B cells and monocytes transfected with recombinant genes from the viral oncogenes E6 and E7 of HPV types 16 and 18 and loaded with α-galactosyl ceramide, a natural killer T (NKT)-cell ligand. BVAC-C can be manufactured overnight following collection of cells from a patient. BVAC-C can potentially target any cancer that is HPV type 16– or 18–positive, including cervical cancer. The vaccine can eliminate immune-system-evading cancer cells by activating innate effector cells and counteracting the exhaustion of effector cells in the tumor microenvironment by inducing NKT cells to release cytokines (22). In a phase I study published in 2020, BVAC-C was well tolerated, exhibited durable anti-tumor activity, and induced a strong immune response in all 11 patients enrolled (23).

Another study explored the combination of BVAC-C with topotecan, a commonly used second-line treatment for cervical cancer (24). The study revealed that a combination therapy produced a significantly stronger antitumor effect compared with topotecan monotherapy, while maintaining the strong immune response induced by BVAC-C. The findings suggest that a combination therapy of BVAC-C and topotecan can increase sensitivity to the action of T and NKT cells, with BVAC-C’s potent induction of the antitumor immune response persisting even in combination therapy.

Here, we present the findings of a phase I/IIa study, which involved open-label administration, dose-escalation, and multiple dosing, to assess the safety, immune response, and effectiveness of BVAC-C as a monotherapy and in combination with topotecan in patients with metastatic or recurrent cervical cancer positive for HPV types 16 or 18 after standard care has failed.

## 2 Methods

### 2.1 Patients and eligibility criteria

This study was a multicenter, phase I/IIa trial investigating the safety and efficacy of BVAC-C in patients with metastatic or recurrent cervical cancers across 6 study sites in Korea. The study protocol was approved by the ethics committee or review board of all participating sites. Informed consent was obtained for all patients, and the trial was carried out in accordance with the Helsinki Declaration on experimentation on human subjects. This study is registered in the Clinical Trials Registry as NCT02866006.

The primary endpoint was overall response rate (ORR), which was defined as the proportion of patients with a complete response (CR) or a partial response (PR), and the 6-month progression-free survival (PFS) rate. Both CR and PR must be confirmed by a tumor evaluation after 28 days. The exploratory objectives were to evaluate the safety and the tolerability of BVAC-C as a monotherapy and in combination with topotecan and to evaluate OS, the disease control rate (DCR), and immune response. All adverse events (AEs) were graded according to the National Cancer Institute Common Terminology Criteria for Adverse Events version 5.0. For multiple occurrences of the same event, that with the worst grade was recorded.

Patients were eligible if they had metastatic or recurrent HPV type 16– or 18–positive cervical cancer and had progressed after 1 prior administration of standard platinum-based chemotherapy with or without bevacizumab. The HPV type was determined using an archived formalin-fixed paraffin-embedded tumor sample or, when HPV typing results of the last 5 years were not available, a newly obtained tumor biopsy sample. Other inclusion criteria were at least 1 measurable lesion according to the Response Evaluation Criteria in Solid Tumors (RECIST) version 1.1; age of 20 years or older; Eastern Cooperative Oncology Group (ECOG) performance status between 0 to 1; life expectancy greater than 3 months; adequate bone marrow function (absolute neutrophil count ≥ 1500/mm^3^ and platelet count ≥ 100,000/mm^3^); adequate renal function (serum creatinine ≤ 2.5 mg/dL); and adequate liver function (transaminase level within 2.5 times the institution’s upper limit of normal). The inclusion criteria for phase I differed, as there was no restriction on the number of prior chemotherapy cycles, and patients with ECOG scores of 0-2 were eligible (23). Incorporating phase I data was justified by the comparable responses and adverse effects observed across various dosage levels (1x10^7^, 4x10^7^, and 1x10^8^ cells/dose).

Patients were excluded if they had neuroendocrine or small-cell carcinoma; a history or signs of brain metastasis; peritoneal carcinomatosis; a history of HIV infection; heart failure, coronary artery disease, or myocardial infarction within 6 months before the screening visit; received any investigational product within 4 weeks before the screening visit; received any vaccine within 4 weeks before the screening visit; received a blood transfusion containing leukocytes within 3 months before the screening visit; received chemotherapy or radiation therapy within 2 weeks before the first administration of the investigational product (BVAC-C); received any of the following medicines within 1 month before the screening visit: chronic steroids (5 days or more), immunosuppressants, immunostimulants, and granulocyte-colony stimulating factor; participated in the clinical trial of immunotherapeutic vaccine within 1 year or in the clinical trial of immune therapy within 6 weeks before the screening visit; a history of serious allergic disease or serious AEs of the drug; or was pregnant or breast-feeding.

### 2.2 Treatment and assessments

The subjects who met the inclusion criteria were sequentially allocated 1:1:1 into 3 groups. In group 1, BVAC-C 1.0 × 10^8^ cells/dose (2mL) was administered repeatedly as an intravenous infusion at day 1 (0 weeks, 4 weeks, and 8 weeks) at 4-week intervals, 3 times in total. In group 2, BVAC-C 5.0 × 10^7^ cells/dose (1mL) was repeatedly administered as an intravenous infusion at day 1 (0 weeks, 4 weeks, 8 weeks, and 12 week) at 4-week intervals, 4 times in total. In group 3, BVAC-C 5.0 × 10^7^ cells/dose (1mL) was repeatedly administered as an intravenous infusion at day 1 (0 weeks, 4 weeks, 8 weeks, 12 weeks) at 4-week intervals, and topotecan 0.75 mg/m^2^ was administered repeatedly as an intravenous infusion at 2 weeks after every BVAC-C administration for 3 days (2 weeks, 6 weeks, 10 weeks, 14 weeks) at 4-week intervals, 4 times in total. Treatment was discontinued in the case of withdrawal of consent, unacceptable toxicity, or investigator decision.

Antitumor efficacy was assessed at 8 weeks using computed tomography or magnetic resonance imaging after first administration of the investigational product and 4 weeks after the last administration of the investigational product. In addition, it was conducted every 8 weeks during the first year and every 12 weeks thereafter. If radiologic imaging indicated progressive disease, a repeat assessment was required at least 4 weeks later to confirm progression.

Safety was monitored throughout the study and for 30 days after treatment discontinuation. All AEs were graded according to the National Cancer Institute’s Common Terminology Criteria for Adverse Events (version 4.0).

### 2.3 Immunological response

#### 2.3.1 *Ex vivo* IFN-γ ELISPOT

Peripheral blood mononuclear cells (PBMCs) were collected and cryopreserved from each participant at the screening visit, every 2 weeks during the treatment period, and at follow-up visits 1 and 2. Thawed PBMCs were incubated with X-VIVO (Lonza, Germany) for more than 16 h at 37°C in 5% CO_2_. PBMCs (2 × 10^5^ cells per well) were subsequently stimulated with 2 µg/mL of 2 different pools of HPV16 E6– and E7–derived peptides (15-mer with 8 overlapping amino acids) for 48 h. After stimulation, spots indicating interferon (IFN)-γ–secreting cells were developed according to the manufacturer’s instructions (R&D Systems, Minneapolis, MN). HPV-specific T-cell responses were calculated by subtracting the spot numbers of peptide-unstimulated T cells from those of peptide-stimulated T cells.

#### 2.3.2 Serum-cytokine analysis

As BVAC-C induces the activation of NK and NKT cells, the levels of IFN-γ and tumor necrosis factor (TNF)-α in the patient blood were analyzed as activation markers of NK and NKT cells. Human plasma samples were obtained at each time point (visit 2, visit 2 + 1 day, visit 4 + 1 day, visit 6 + 1 and visit 8 + 1 day), and the levels of each cytokine were measured with Milliplex technology (Millipore, Burlington, MA), enzyme-linked immunosorbent assay kits (R&D Systems, Minneapolis, MN) using a Luminex 200 microbead analyzer (Millipore, Burlington, MA), or a Multiskan SkyHigh microplate spectrophotometer (Thermo Fisher, Waltham, MA) according to the manufacturer’s instructions.

### 2.4 Statistical analysis

To determine the maximum tolerable dose in phase I of the study, 3 subjects for each dose group were enrolled based on the standard 3 + 3 plan (25). For phase IIa to evaluate the efficacy of monotherapy and combination therapy, 7 subjects for each set group were enrolled. The total number of the subjects of phase I/IIa trial ranged from 30 to 39.

Descriptive statistics for each dose group as well those for all subjects were used to assess the safety and tolerability endpoints in all patients who received at least 1 dose of BVAC-C. Efficacy was assessed in all patients who had measurable disease at baseline according to RECIST v1.1 and who received all doses of BVAC-C. Immunogenicity endpoints were assessed in the intent-to-treat population, defined as all subjects who received any amount of study treatment and who had at least 1 post-baseline immunological evaluation. Statistical analysis was performed using R version 4.0.0.

## 3 Results

### 3.1 Patients

**Figure 1:**
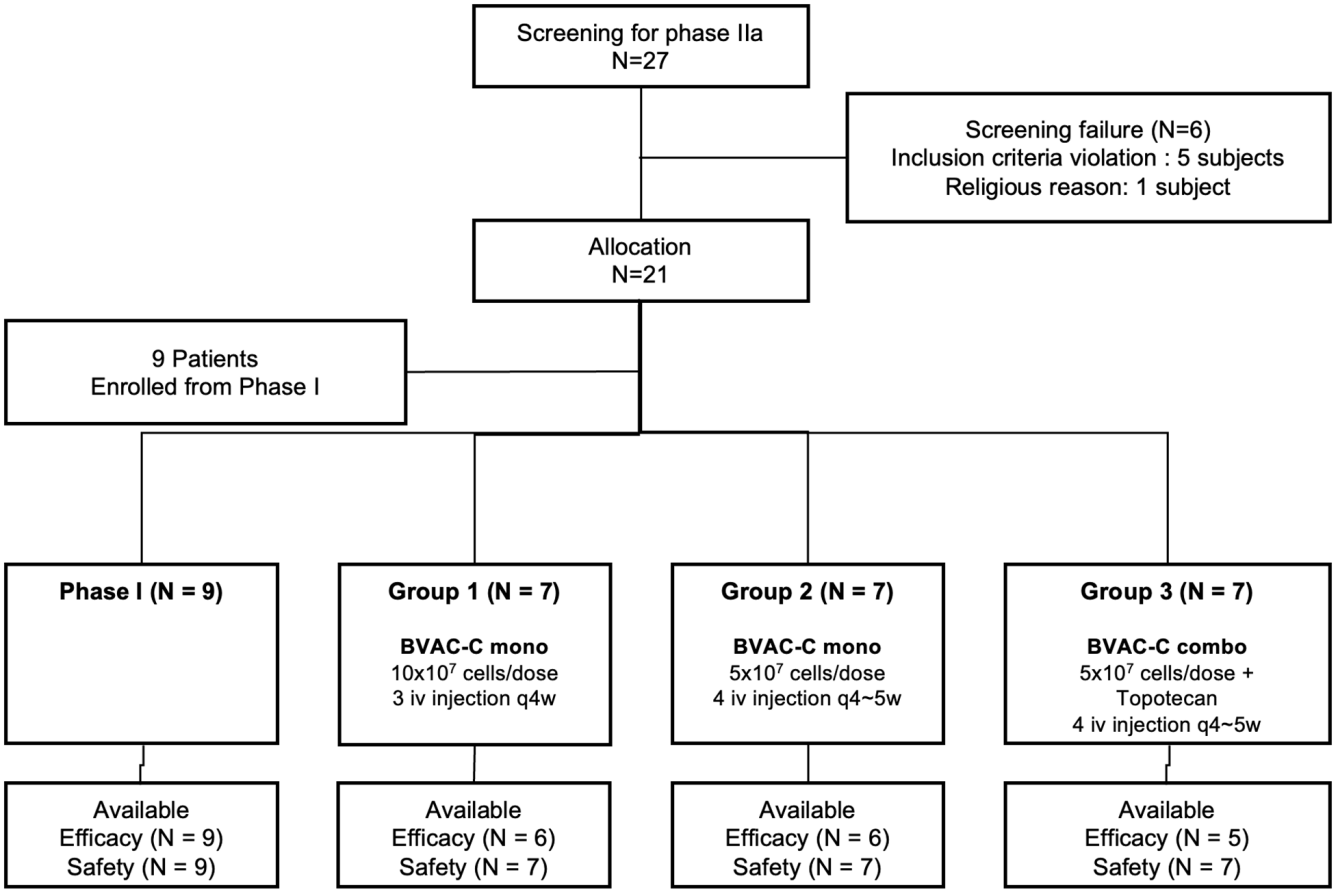
Study flowchart.

Between September 2018 and October 2019, of 27 patients screened, 21 were enrolled in this phase IIa study. Six patients were ineligible, with the most common reasons being violation of inclusion criteria (n = 5). With the addition of 9 patients enrolled during the phase I study, 30 were available for analysis (**Figure 1**). As of November 2022, the median follow-up duration was 13 months (range, 3.7–46.9). Four patients dropped out before completion of scheduled treatment, most commonly due to withdrawal of consent (n = 3, 10.0%).

Patient characteristics and baseline demographics according to group are described in **Table 1**. Median age at enrollment was 48.5 years (range, 30–68). Most patients (66.6%) had an ECOG performance score of 1, and almost all had squamous cell carcinoma (76.7%) and were HPV 16–positive (90.0%). Eleven (36.7%) had received primary chemoradiation and 19 (63.3%) had received primary surgery. All patients presented with metastatic disease, which was most frequently located in the lymph node (*n* = 18; 60.0%), lung (*n* = 10; 33.3%), and pelvis (*n* = 6; 20.0%). There was no consequential difference between the groups.

**Table 1:** Baseline patient characteristics of the cohort.

	BVAC-C (x3) (N = 16)	BVAC-C (x4) (N = 7)	BVAC-C+Topotecan (N = 7)	p
Age	49.7 ± 8.8	51.0 ± 9.1	45.4 ± 14.8	0.574
ECOG				0.554
0	3 (18.8%)	2 (28.6%)	2 (28.6%)	
1	10 (62.5%)	5 (71.4%)	5 (71.4%)	
2	3 (18.8%)	0 ( 0.0%)	0 ( 0.0%)	
HPV				0.597
16	14 (87.5%)	7 (100.0%)	6 (85.7%)	
18	2 (12.5%)	0 ( 0.0%)	1 (14.3%)	
Histology				0.798
AD	4 (25.0%)	2 (28.6%)	1 (14.3%)	
SCC	12 (75.0%)	5 (71.4%)	6 (85.7%)	
Size (cm)	4.1 ± 2.5	3.3 ± 2.0	2.5 ± 1.0	0.237
Primary_treatment				0.690
CCRT	7 (43.8%)	2 (28.6%)	2 (28.6%)	
Surgery	9 (56.2%)	5 (71.4%)	5 (71.4%)	
Dx to BVAC (mo)	36.6 ± 41.6	40.0 ± 19.9	41.3 ± 29.7	0.953
Tumor site				
Pelvis	4	1	1	
Lymph node	11	4	3	
Lung	5	2	3	
Response				0.774
CR	1 ( 6.7%)	0 ( 0.0%)	0 ( 0.0%)	
PR	2 (13.3%)	2 (33.3%)	0 ( 0.0%)	
SD	5 (33.3%)	2 (33.3%)	2 (40.0%)	
PD	7 (46.7%)	2 (33.3%)	3 (60.0%)	

ECOG PS, Eastern Cooperative Oncology Group performance status; SCC, squamous cell; AD, adenocarcinoma; LNs, lymph nodes; Dx, Diagnosis; PD, progressive disease; CR, complete response; PR, partial response; SD, stable disease.

### 3.2 Tumor response

Twenty-six patients were evaluable for clinical response (**Table 1**). An objective response was observed in 5 patients, and the ORR was 19.2% (95% CI 4.1–34.4); a single patient (3.8%) achieved a CR, and 4 (15.4%) achieved a PR as their best overall response. Overall, the DCR was 53.8% (95% CI 34.7–73.0) and was not associated with any treatment group (**Table 2**). The DCR appeared to be higher in patients with a favorable ECOG score and longer diagnosis to the BVAC-C interval, although these factors were not statistically significant (p = 0.06 and p = 0.176, respectively).

**Table 2:** Disease control rate by clinical factors.

	PD	CR/PR/SD	p
Group			0.676
BVAC-C (x3)	7 (58.3%)	8 (57.1%)	
BVAC-C (x4)	2 (16.7%)	4 (28.6%)	
BVAC-C+Topotecan	3 (25.0%)	2 (14.3%)	
ECOG			0.061
0	1 (8.3%)	5 (35.7%)	
1	8 (66.7%)	9 (64.3%)	
2	3 (25.0%)	0 ( 0.0%)	
HPV			1.000
16	11 (91.7%)	13 (92.9%)	
18	1 ( 8.3%)	1 ( 7.1%)	
Histology			0.802
AD	2 (16.7%)	4 (28.6%)	
SCC	10 (83.3%)	10 (71.4%)	
Size	3.8 ± 2.5	3.4 ± 2.2	0.677
Primary treatment			0.589
CCRT	3 (25.0%)	6 (42.9%)	
Surgery	9 (75.0%)	8 (57.1%)	
Dx to BVAC (mo)	28.7 ± 11.3	47.7 ± 46.5	0.176
Plasma IFN-γ fold change			0.937
< 1	1 (8.3%)	0 (0%)	
> 1 (increased)	11 (91.7%)	14 (100%)	
Plasma TNF-α fold change			0.394
< 1	2 (16.7%)	0 (0%)	
> 1 (increased)	10 (83.3%)	14 (100%)	
HPV type-matched T cell response^a^			0.443
Positive	10 (83.3%)	8 (61.5%)	
Negative	2 (16.7%)	5 (38.5%)	

PD, progressive disease; CR, complete response; PR, partial response; SD, stable disease.

a T cells from SD patient S31 were not analyzed.

**Figure 2:**
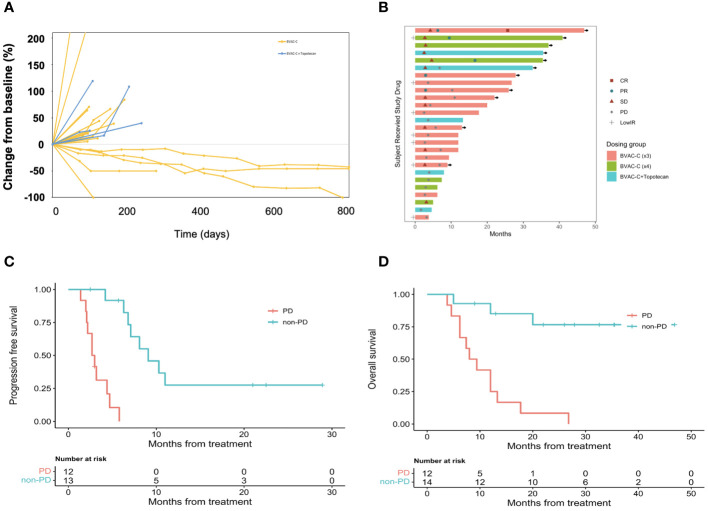
Antitumor activity of BVAC-C. **(A)** Time on treatment, time to best response, and duration of response. Continuation of response despite immunotherapy discontinuation is an important efficacy metric. Symbols along each bar represent relevant clinical events, such as progressive disease (PD), stable disease (SD), partial response (PR), or low immune response (LowIR). **(B)** Best percentage change in target lesion size from baseline. Dotted lines at 20% and −30% indicate the percentage change from baseline and represent progressive disease and partial response, respectively, under RECIST v1.1. **(C)** Progression free survival according to response. **(D)** Overall survival according to response.

A decrease from baseline in the diameter of the target lesions is shown in **Figure 2A**. A prolonged decrease in tumor size was seen in 3 patients. The combination of BVAC-C and topotecan showed more progressive disease and further study is needed. A swimmer plot is presented in **Figure 2B**. We combined the phase I data with the phase II study (Arm 1, BVAC-C cycle of 3) due to the absence of discernible differences in efficacy between the two groups.

Of the 26 patients with at least 1 post-baseline imaging assessment, 7 (26.9%) experienced a reduction in target lesion size from baseline, and 5 (19.2%) recorded a reduction ≥ 30%. The median DOR was 7.5 months (95% CI 7.1–not reported). Median PFS was 5.8 months (95% CI 4.2–10.3); the 6- and 12-month PFS rates were 48.7% (95% CI 31.9–74.2) and 14.6% (95% CI 5.2 – 41.2), respectively (**Figure 2C**). Median OS was 17.7 months (95% CI 12.0–not reported); and the 6- and 12-month OS rates were 88.5% (95% CI 77.0–100.0) and 56.8% (95% CI 40.5– 79.8), respectively (**Figure 2D**).

### 3.3 Adverse events

The most common AEs are summarized in **Table 3**. Some of the most common treatment-emergent adverse events (TEAEs) were pyrexia (n = 20; 66.7%), myalgia (n = 6; 20.0%), nausea (n = 4; 13.3%), and vomiting (n = 4; 13.3%). Most TEAEs were grade 1 and manageable. A grade 3 TEAE, pyrexia, was observed in only 1 patient.

**Table 3:** Treatment-related adverse events of any grade observed in the study (*n* = 30).

Adverse events	BVAC-C x3 (*n* = 16)			BVAC-C x4 (*n* = 7)			BVAC-C+Topotecan (*n* = 7)			Total, No. (*N* = 30, %)		
	G1	G2	G3	G1	G2	G3	G1	G2	G3	G1	G2	G3
Pyrexia	11	1	0	1	2	0	4	0	1	16 (53)	3 (10)	1 (3)
Chills	3	0	0	0	0	0	0	0	0	3 (10)	0	0
Fatigue	1	0	0	0	0	0	0	0	0	1(3)	0	0
Myalgia	4	1	0	0	0	0	0	1	0	4 (13)	2(7)	0
Diarrhea	0	1	0	0	0	0	0	0	0	0	1(3)	0
Nausea	3	0	0	0	0	0	1	0	0	4 (13)	0	0
Vomiting	2	0	0	0	1	0	1	0	0	3 (10)	1(3)	0
Cytokine release syndrome	0	1	0	0	0	0	0	0	0	0	1(3)	0
Headache	3	0	0	0	0	0	0	0	0	3 (10)	0	0
Dizziness	1	0	0	0	0	0	0	0	0	1 (3)	0	0

No patient discontinued the trial due to unacceptable toxicities, and no dose-limiting toxicities occurred. No deaths that were possibly related to the study therapy were noted; the deaths reported were related to progression of underlying tumor. There were no infusion-related reactions. Immune-related adverse events (irAEs) were observed in 20 patients, including 1 who experienced grade 2 cytokine release syndrome.

### 3.4 Immunogenicity

#### 3.4.1 Plasma-cytokine analysis

**Figure 3:**
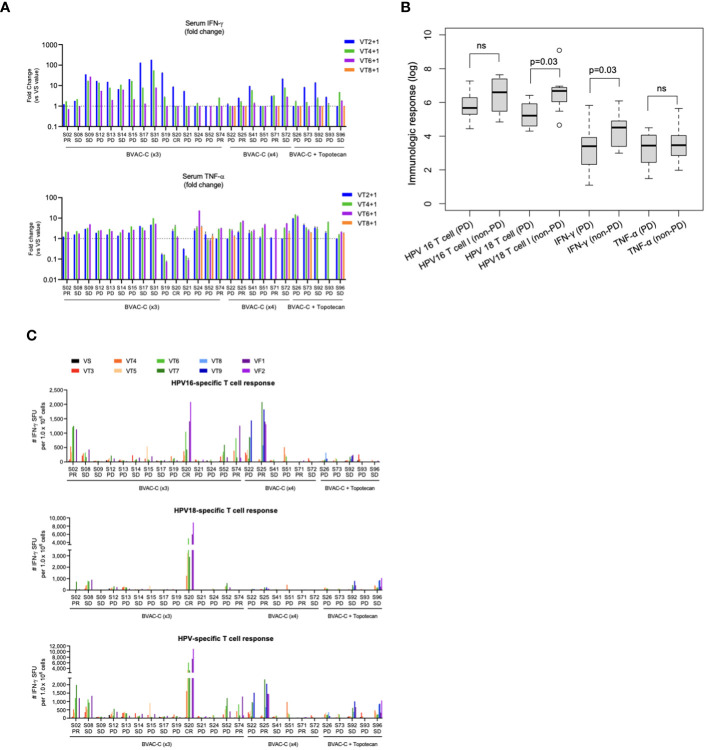
Immunological response. **(A)** Fold-changes in the levels of serum INF-γ and TNF-α at each timepoint against visit 2. **(B)** Boxplot of immunologic markers and responses. **(C)** HPV-specific T-cell spots at each timepoint. ns, not significant.

Theoretically, BVAC-C interacts with a patient’s NKT cells when administered, and the cell types activate each other, which results in differentiation of BVAC-C into antigen-presenting cells and release of cytokine from NKT cells. In addition, IFN-γ released by NKT cells subsequently induces NK cell activation, resulting in the release of additional IFN-γ and TNF-α. The plasma samples were collected from each patient one day after BVAC-C administration to evaluate the activation of NK and NKT cells. Most plasma samples showed elevated levels of IFN-γ and TNF-α. Compared with the analysis results of visit 2 plasma, the levels of IFN-γ and TNF-α on one day after BVAC-C administration were elevated in 25 and 24 of 26 patients, respectively (**Tables 2**, **4**). The increases in IFN-γ level varied from 0.72-fold (S02) to 181.8-fold (S31) after BVAC-C administration while those of TNF-α levels varied from 2.2-fold (S02) to 23.6-fold (S26) above baseline (**Figure 3A**). The fold-increase in IFN-γ was lower in patients with progressive disease (**Figure 3B**).

**Table 4:** Best overall response as assessed by the investigator review according to immune response induced by BVAC-C administration.

Patient ID	HPV type	Best overall response	HPV16-specific T cell ^a^	HPV18-specific T cell ^a^	IFN-γ ng/L^b^	TNF-α ng/L^b^
S02	16	PR	1250	737.5	134.5	18.4
S08	16	SD	432.5	905	110	62.2
S09	16	SD	–	–	110.2	48.2
S12	16	PD	220	–	45.1	49.1
S13	16	PD	-	322.5	40.8	61.4
S14	16	SD	232.5	105	29.7	53.1
S15	16	PD	535	372.5	56.2	89.7
S17	18	SD	-	-	321.3	56.9
S31	16	SD	NA	NA	441.9	281.7
S19	16	PD	200	80	338.5	76.2
S20	16	CR	2085	8880	70.2	15.7
S21	16	PD	85	-	42.7	37.6
S22	16	PD	1440	100	9.8	11.8
S24	16	PD	-	130	10.6	4.4
S25	16	PR	2083.3	240	19.9	27.2
S26	18	PD	323.3	260	13.0	54.2
S41	16	SD	-	-	75.2	11.8
S51	16	PD	513	463	<LOD	8.8
S52	16	PD	597	613.7	<LOD	12.9
S71	16	PR	-	-	25.3	17.3
S72	16	SD	126.7	-	172.9	29.3
S73	16	PD	138.3	121.7	66.9	11.2
S74	16	PR	1260.3	-	19.9	56.6
S92	16	SD	246.7	796.7	111.9	34.6
S93	16	PD	260.3	73.7	22.1	25.8
S96	16	SD	-	1055	36.7	7.2

^a^HPV type-specific T cell spots/10^6^ PBMC, which was collected at the maximum SFU time-point. NA, No Assessment. ^b^The highest serum IFN-γ or TNF-α concentration measured one day after each BVAC-C administration.

#### 3.4.2 *Ex vivo* IFN-γ ELISPOT

The HPV-specific T-cell response was analyzed every 2 weeks during the clinical study. The number of IFN-γ spots was higher after BVAC-C treatment in most patients compared with the baseline control (**Figure 3C**). In 5 patients among the 25 evaluated, the spot number of HPV type–matched and E6/E7-specific T cells exceeded 1,000, which was 0.1% of peripheral blood T cells (S02, S20, S22, S25, and S74; **Table 4**). The strongest HPV-specific T-cell responses were observed in patient S20, who achieved a CR and showed high induction of both HPV type 16– and 18–specific T-cell responses. Additionally, the patient’s HPV type–matched and E6E7-specific T-cell responses were strongly induced in patients S02, S25, and S74, who had PRs.

## 4 Discussion

The study produced promising results for BVAC-C as a therapeutic strategy for metastatic or recurrent HPV type 16– or 18–positive cervical cancer. BVAC-C demonstrated promising antitumor activity, with an ORR of 19.2% and a DCR of 53.8%, as well as the ability to elicit innate and adaptive immune responses. However, the addition of topotecan did not increase antitumor activity. This study demonstrated the potential efficacy of BVAC-C, which consists of autologous B cells and monocytes transfected with recombinant viral gene, loaded with NKT-cell ligand, and can be manufactured overnight. In the context of clinical practice, our findings underscore the potential of BVAC-C as a viable treatment option with a manageable safety profile for patients with HPV 16– or 18–positive cervical cancer. Moving forward, future studies should focus on identifying biomarkers of response to further optimize patient selection and treatment outcomes in this setting. An ongoing investigation of BVAC-C in combination with a PD-L1 inhibitor could further explore its clinical efficacy.

Previous studies have tested various therapeutic vaccine candidates for cancer treatment, including chimeric virus-like particles, dendritic cell (DC) vaccines, fusion proteins, peptides, attenuated viruses, and recombinant viruses (15, 18, 19). BVAC-C, a B cell– and monocyte-based vaccine, offers practical advantages over DC vaccines. Unlike DCs, B cells and monocytes are abundant in peripheral blood and easily collected, thereby enhancing manufacturing efficiency. The BVAC-C drug product can be manufactured in a single day in a facility with good manufacturing practices and can be stored frozen for multiple administrations. The clinical trial demonstrated that BVAC-C is highly immunogenic and exhibits antitumor efficacy, supporting the modes of action suggested by animal studies. Previous studies have shown that BVAC loaded with an invariant NKT cell ligand, α-galactosyl ceramide, can elicit diverse antitumor immune responses and overcome immune-system-evading mechanisms of cancer cells in the tumor microenvironment (20–22, 26, 27).

Cervical cancer treatment requires new agents with reduced toxicity. The AEs observed in this study, including treatment-related and immune-related AEs, were mostly grade 1 and 2, consistent with other immunotherapies tested in different studies (8, 9, 12). Fever and myalgia were the most common AEs but were manageable. Overall, the safety profile of BVAC-C in this trial indicates that it could be a suitable treatment option for patients who have undergone multiple lines of therapy.

BVAC-C can induce the activation of NKT cells via CD1d and α-galactosyl ceramide complexes on the cell surfaces of the drug product, and activated NKT cells can elicit the activation of NK cells. As the activated NK and NKT cells secret inflammatory cytokines, we used IFN-γ and TNF-α as markers of NK- and NKT-cell activation. While the level of cytokine secretion varied at each time point, the majority of patients showed elevated levels of both cytokines within 1 day following BVAC-C treatment. The activation of HPV E6/E7–specific T cells was also observed in most participants during the clinical study. Overall, the types and magnitudes and of immune responses induced by BVAC-C varied, but immune responses were observed in all patients. However, we did not observe a significant correlation between immunogenicity and clinical responses in our analysis. Nonetheless, it is worth noting that a potential correlation may still exist. For instance, Patient S20, who achieved a complete response (CR), exhibited the most robust HPV-specific T-cell response among all participants in the clinical trial. Additionally, the patient’s HPV type–matched and E6E7-specific T-cell responses were strongly induced in patients S02, S25, and S74, who had PRs. This suggests that cervical cancer can be controlled through strong HPV-specific T-cell responses by BVAC-C administration. However, the progressive disease patient, S22, showed a poor clinical response despite high T-cell immunogenicity, possibly due to other factors, such as the patient health condition, anti-cancer drugs used before BVAC-C treatment, and cancer immunosuppression. It is necessary to identify other factors affecting BVAC-C efficacy.

The unexpected finding of reduced antitumor efficacy with the combination of topotecan and BVAC-C, despite the small sample size, warrants attention. Numerous studies have demonstrated that cytotoxic agents can modulate tumor cell phenotype, rendering them more susceptible to immune attack and promoting immunogenic cell death. This initiates a series of events involving dendritic cell activation and cytotoxic T lymphocyte stimulation, thereby enhancing their ability to target tumor cells (28, 29). Additionally, topotecan exhibits the ability to influence various immune regulatory elements, induce shifts in leukocyte populations, and synergize with vaccines to bolster immune responses against tumor-associated antigens. Despite the promising outcomes observed in preclinical and clinical studies, it is essential to acknowledge potential limitations. One significant concern is the potential for chemotherapy-induced immunosuppression, which may compromise the efficacy of therapeutic vaccines by dampening immune responses or altering the tumor microenvironment in ways that favor tumor growth or treatment resistance (30). Several studies investigating vaccine-chemotherapy combinations have underscored the importance of carefully timing and dosing chemotherapy in relation to vaccine administration for optimal efficacy (31, 32). Furthermore, administering chemotherapies at doses considered suboptimal for primary treatment could inadvertently facilitate tumor adaptation and resistance. Thus, while the combination of topotecan and therapeutic vaccines shows potential for enhancing antitumor immunity, careful consideration of potential negative effects and strategic treatment optimization are imperative for maximizing therapeutic outcomes.

Our study has limitations, including a small sample size and the absence of a control arm, which may have affected the robustness of the results. Additionally, a high drop-out rate and the lack of a predictor of prolonged response are research shortcomings.

In conclusion, BVAC-C demonstrated durable antitumor activity in patients with recurrent cervical cancer positive for HPV 16/18, and its manageable safety profile makes it a meaningful option in the second-line setting. Based on these promising results, we have initiated a phase II multicenter clinical trial of BVAC-C in combination with PD-L1 inhibitors for women with recurrent cervical cancer. Although our study reported relatively high response rates, further confirmatory data from larger randomized trials will be required to identify potential biomarkers of response.

## Data availability statement

The raw data supporting the conclusions of this article will be made available by the authors, without undue reservation.

## Ethics statement

The studies involving humans were approved by Samsung Seoul Hospital, Samsung Medical Center. The studies were conducted in accordance with the local legislation and institutional requirements. The participants provided their written informed consent to participate in this study.

## Author contributions

CC: Conceptualization, Data curation, Formal analysis, Methodology, Visualization, Writing – original draft, Writing – review & editing. BK: Writing – original draft, Writing – review & editing. JL: Writing – original draft, Writing – review & editing. DB: Writing – original draft, Writing – review & editing. EK: Writing – original draft, Writing – review & editing. DC: Writing – original draft, Writing – review & editing. YK: Writing – original draft, Writing – review & editing. KK: Writing – original draft, Writing – review & editing. JK: Writing – original draft, Writing – review & editing. HK: Writing – original draft, Writing – review & editing. YK: Writing – original draft, Writing – review & editing. JL: Writing – original draft, Writing – review & editing. ML: Writing – original draft, Writing – review & editing. TO: Writing – original draft, Writing – review & editing. BS: Writing – original draft, Writing – review & editing. IJ: Writing – original draft, Writing – review & editing. MP: Writing – original draft, Writing – review & editing. WK: Writing – original draft, Writing – review & editing. CK: Writing – original draft, Writing – review & editing.
